# Association of ABO and RhD blood groups with the risk of HIV infection

**DOI:** 10.1371/journal.pone.0284975

**Published:** 2023-04-26

**Authors:** Genevieve Jacobs, Karin Van den Berg, Marion Vermeulen, Ronel Swanevelder, Brian Custer, Edward L. Murphy

**Affiliations:** 1 South African National Blood Service, Johannesburg, South Africa; 2 Vitalant Research Institute, San Francisco, California, United States of America; 3 University of California San Francisco, San Francisco, California, United States of America; United States Environmental Protection Agency, UNITED STATES

## Abstract

Naturally occurring antibodies against ABO antigens present in human sera have been shown to neutralize ABO-expressing HIV in vitro. We investigated associations between ABO and RhD blood groups and HIV infection among blood donors from all blood collection centers in eight of South Africa’s nine provinces. Whole blood donations collected from first time donors between January 2012 and September 2016 were tested for HIV RNA by nucleic acid testing and HIV antibody using third generation serology assays. ABO and RhD blood types were determined using automated technology. Odds ratios for the association between HIV positivity and ABO and RhD phenotypes were calculated using multivariable logistic regression analysis. We analyzed 515,945 first time blood donors and the overall HIV prevalence was 1.12% (n = 5790). After multivariable adjustment, HIV infection was weakly associated with RhD positive phenotype (OR = 1.15, 95% CI 1.00–1.33) but not with ABO blood group. The observed association with RhD positive phenotype was marginal and likely due to residual confounding by racial group but could serve to generate hypotheses for further studies.

## Introduction

In sub-Saharan Africa, HIV infection and AIDS continue to be a burden on public health care as the HIV pandemic approaches its semi-centennial anniversary. Remarkable multi-disciplinary advances in detection, prevention and treatment of AIDS have been made including the discovery of its causative agent, evolved detection methods, improvements in patient care and drug therapy to social and behavioral interventions but HIV remains a challenge with no cure forthcoming. Blood transfusion was one of the first documented routes of HIV transmission [1, 2] with measures to protect the blood supply culminating in the recommendation for mandatory HIV testing of donor blood prior to transfusion [3–5]. The main role of the ABO histo-blood group system in transfusion science is the prevention of transfusion reactions through blood group compatibility testing, but it has also been reported that blood groups are linked to infectious disease susceptibility, including viral diseases [6, 7]. In laboratory studies ABO blood group antibodies were able to neutralize HIV in vitro [8]. Neil et al demonstrated that HIV-1 infected lymphocytes possessing cell surface ABO blood group oligosaccharides allow the integration of these host-derived antigens into the viral envelope during the budding process of viral replication. Moreover, naturally occurring antibodies in human sera neutralized corresponding ABO antigens carried by the HIV virion via complement activation [8]. These findings suggest that ABO blood groups may play a role in reducing HIV susceptibility, through antibody-mediated neutralization between discordant blood groups expressed by the infecting virus from the primary host and the subsequent host. Recent research on blood groups and SARS-COV-2 infection have reported similar interactions between ABO incompatibility and decreased risk of COVID-19 transmission, suggesting neutralization by naturally occurring anti-ABO antibodies [9, 10]. The relationship between blood groups and HIV infection have been explored with varying results and stands contended [11, 12]. The aim of this study was to investigate whether ABO and RhD blood groups are associated with HIV infection in South African blood donors.

## Materials and methods

### Setting

South Africa has an estimated population of 60 million people living across nine provinces. The prevalence of HIV infection is estimated at 13.68% [13]. The South African National Blood Service (SANBS) collects blood from voluntary, non-remunerated blood donors from eight of its nine provinces (excluding the Western Cape). Donors can donate blood at permanent collection centres (fixed site) or temporary venues around the country (mobile site). The prevalence of HIV in SANBS first-time donors is 1.1% [14].

The South African government historically classified its population into four major racial groups: Black (80.9%), Coloured (8.8%) White (7.8%) and Indian/Asian (2.6%) [13]. ‘Coloured’ is a historical political term used in South Africa which refers to a phenotypically diverse population of individuals with varied ancestry including African, European and Southeast Asian origin [15]. To date, South African individuals are still required to self-report race on official documents for statistical purposes. This includes the process of donating blood and SANBS collects data on race for this reason, in addition to expediting the search for phenotypically matched blood when required. In this study, race, in addition to other demographics was included to address potential confounding.

### Study design and population

We performed a cross-sectional study of first-time blood donors at SANBS between January 2012 and September 2016. All first-time blood donors who had donated a unit of whole blood at any of the blood collection centres (mobile and fixed site) in every province served by SANBS were included in the study. Demographic characteristics were recorded on a donor history questionnaire that was completed by all persons presenting to donate blood. Data retrieved from the SANBS donor information database included the following variables: gender, age, race, collection centre type (fixed or mobile), HIV, HBV and HCV status, and geographic region (province).

This study was approved by the SANBS Human Research Ethics Committee certificate number 2011/07. Data for this study were extracted from operational databases and laboratory testing for which blood donors gave consent at the time of donation. Additional consent was waived by the ethics committee.

### Laboratory testing

Each donor was blood typed for ABO and RhD using the PK 7300 (Beckman Coulter, USA). Inconclusive results from the PK7300 were confirmed by manual forward and reverse blood group agglutination testing using Anti-A, Anti-B, Anti-AB and anti-D sera, and corresponding red cell antigen reagents which were manufactured in-house.

All donations were routinely screened in parallel for HIV, HBV and HCV by both serological and individual donation nucleic acid amplification testing (ID-NAT). Each donor was screened for HIV 1 and 2 antibodies, HBV surface antigen (HBsAg) and HCV antibodies using the Prism anti-HIV, HBsAg and anti-HCV assays (Abbott, Delkenheim, Germany). Samples found reactive on initial Prism testing were repeated twice on the same platform. Each donor was tested by ID-NAT using the Grifols Ultrio Plus assay, Tigris Platform (Grifols, Barcelona, Spain). Samples found reactive on the Ultrio Plus assay were repeated in duplicate with additional discriminatory testing using the Grifols dHIV, dHBV and dHCV assays on the Tigris platform. No further confirmatory testing was performed on samples that tested positive on both Serology and NAT screening.

Confirmatory testing was performed on Serology/NAT discordant results. Confirmatory serology testing for HIV was performed by Geenius immunoblot (Bio-Rad, Hercules, CA), HBV by HBsAg neutralization (Roche, Pleasanton, CA, USA) and HCV by line immunoassay InnoLIA (Innogenetics, Ghant, Belgium). A donor was classified as being HIV positive in any of the following scenarios: both anti-HIV and ID-NAT tests were positive; or a repeat positive serology screen and confirmatory serology testing; or repeatedly positive NAT testing.

### Statistical analysis

All demographic variables, donation and laboratory data were captured electronically in the blood establishment computer system, from where all transactional data was extracted, transformed and loaded into a SQL based Data Warehouse. ABO and RhD were analyzed as separate predictors. Unadjusted odds ratios with 95% confidence intervals were calculated using logistic regression. Adjusted odds ratios were then obtained from multivariate logistic regression models including potential confounding variables such as race, age, sex, province, donation site and HBV and HCV co-infection. Finally, we evaluated interaction terms but none were significant. Statistical analysis was performed using SAS (SAS 9.3, SAS Institute).

## Results

There were 515 397 first time donors who donated a unit of whole blood between January 2012 and September 2016. The majority of participants were under the age of 30 (72%), Black (54%) and female (56%) and had donated blood at a mobile donation site (84%) (Table 1). The Gauteng province (44%) of South Africa had the largest share of donors and the Northern Cape (3%) the smallest share. There was an unequal distribution of ABO blood groups; the most prevalent being blood group O (45%), followed by A (33%), B (17%) and AB (5%). Most donors were RhD Positive (93%) and less than a tenth were RhD Negative (7%).

**Table 1:** Demographic characteristics and multivariable analysis of 515 397 first time donors between January 2012 and December 2016 showing the effect of donor demographics and ABO and RhD blood group on HIV positive status.

Effect	First-time donors (n)	HIV Positives (n)	Prevalence (%)	Multivariable Odds Ratio	(95% Confidence Interval)
**Gender**					
Male	228059	1776	0.78%	1	—
Female	287330	4014	1.40%	1.72	1.62–1.82
Missing	8	0	0.00%	*	*
**Age group**					
< = 19y	238212	1430	0.60%	1	—
20-24y	74630	1082	1.45%	2.1	1.94–2.28
25-29y	59994	1089	1.82%	2.73	2.52–2.97
30-39y	74075	1378	1.86%	3.08	2.85–3.32
> = 40	68486	811	1.18%	2.59	2.37–2.84
**Race**					
White	158149	97	0.06%	1	—
Indian/Asian	36475	35	0.10%	1.33	0.90–1.96
Black	277458	5427	1.96%	27.85	22.80–34.09
Coloured	28132	128	0.45%	6.81	5.21–8.89
Unknown	15183	103	0.68%	9.49	7.18–12.55
**Donation Site**					
Fixed	80031	695	0.87%	1	—
Mobile	435366	5095	1.17%	1.25	1.15–1.36
**HBV**					
Negative	511964	5630	1.10%	1	—
Positive	3433	160	4.66%	2.27	1.93–2.67
**HCV**					
Negative	515228	5782	1.12%	1	—
Positive	169	8	4.73%	4.43	2.13–9.19
**Province**					
Gauteng	229055	2281	1.00%	1	—
Eastern Cape	48529	479	0.99%	1.18	1.07–1.31
Free State	27231	316	1.16%	1.38	1.22–1.55
Kwa-Zulu Natal	105281	1254	1.19%	1.53	1.43–1.64
Limpopo	21090	216	1.02%	0.85	0.74–0.98
Mpumalanga	47007	1002	2.13%	2.18	2.02–2.36
North West	24144	147	0.61%	0.92	0.78–1.09
Northern Cape	13054	95	0.73%	1.32	1.07–1.63
Missing	6	0	0.00%	*	*
**RhD**					
Negative	36704	201	0.55%	1	—
Positive	478469	5576	1.17%	1.15	1.00–1.33
Missing	224	13	5.80%	*	*
**ABO**					
A	168642	1763	1.05%	1	—
AB	25329	295	1.16%	1.03	0.91–1.17
B	88980	1078	1.21%	1	0.93–1.08
O	232223	2641	1.14%	0.96	0.91–1.02
Missing	223	13	5.83%	*	*

**Fig 1:**
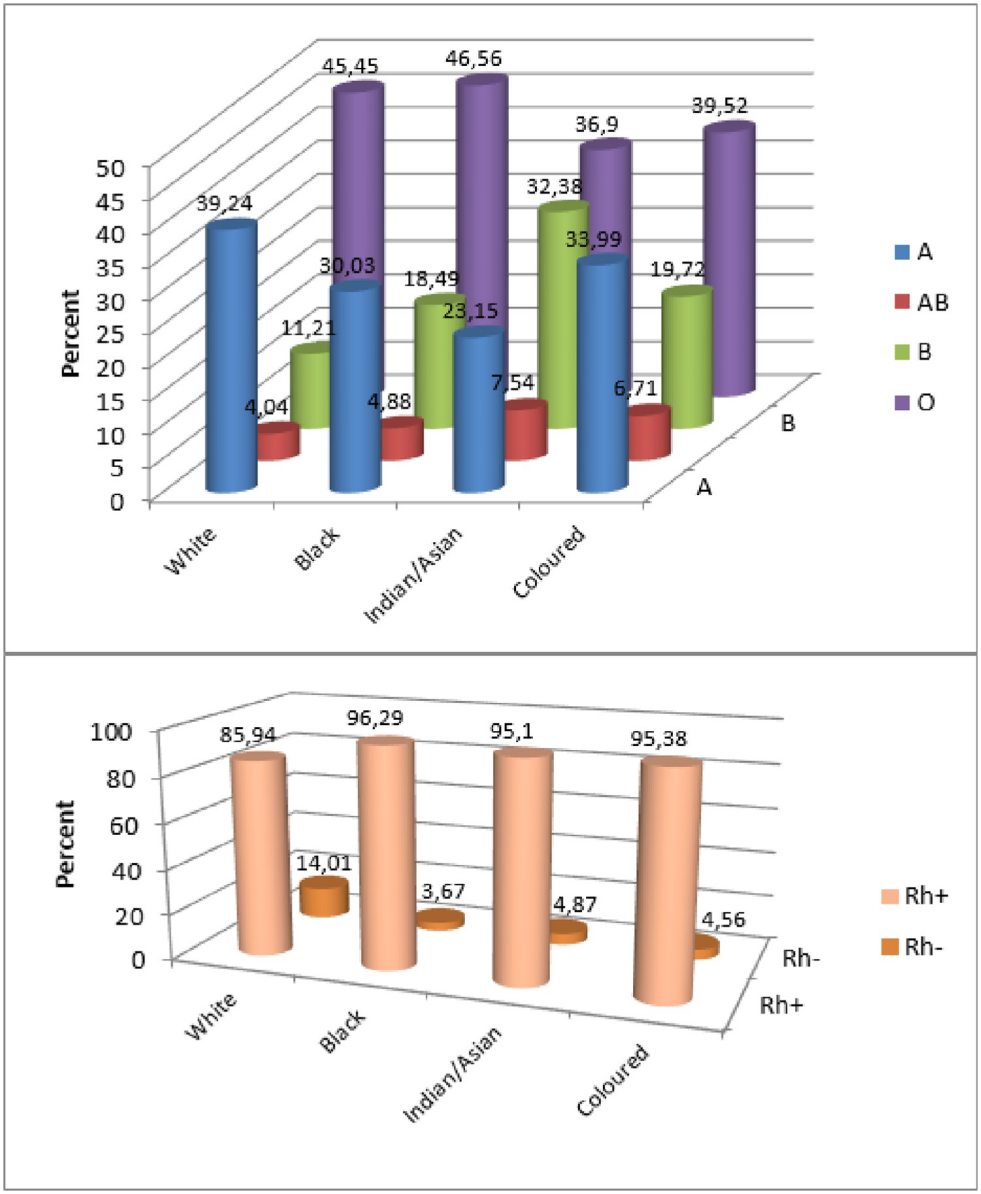
ABO and RhD blood group frequencies by race.

Blood group frequencies amongst the four race groups followed a similar ordinal pattern but with notable differences by race (Fig 1). For example, RhD positivity was much higher in the Black compared to White race group and blood group AB was higher in the Indian/Asian and Coloured groups compared to other racial groups. Blood group A was less common in the Black and Indian/Asian versus White and Coloured race group, while blood group B was more common in Indian/Asian, Black and Coloured vs White race groups.

Of the 515 397 first time donors tested, 5790 (1.12%) were HIV positive, 3433 (0.67%) were HBV positive and 169 (0.03%) were HCV positive. In the unadjusted analysis HIV infection was associated with blood groups AB (OR = 1.12, 95% CI 0.99–1.26), B (OR = 1.16, 85% CI 1.08–1.25) and O (OR = 1.09, CI 95% 1.03–1.16) compared to blood group A. HIV positivity was also strongly associated with RhD positive phenotype (OR = 2.14, 95% CI 1.86–2.47). However, after adjusting for race, age, sex, province, donation site, HBV and HCV status in the multivariable model, HIV infection had a weak and statistically borderline association with RhD positivity (adjusted OR = 1.15, 95% CI 1.00–1.33) but not with any of the ABO blood groups (Table 1). HIV was also significantly associated with female sex, age 20 years or older, Black and Coloured race, donation at a mobile collection site, hepatitis B and C positivity and donation in several provinces other than Gauteng. We also examined models with interaction terms between race group and ABO phenotype and between race group and RhD phenotype but none were significant.

## Discussion

Our findings do not support our hypothesis that ABO blood group was associated with HIV infection although we did find a borderline association of HIV seropositivity with RhD positive phenotype of unclear significance. As expected, HIV infection was also associated with demographic characteristics, particularly being young, Black and female. This is consistent with other studies of HIV in the South African general population [13] with some citing socioeconomic factors as a possible link [16].

Overall, donors with RhD positive phenotype had higher HIV prevalence than those who were RhD negative but we suspect that this finding may be attributed to residual confounding. RhD phenotype differed substantially among racial groups and the association between RhD and HIV was greatly diminished after adjustment for race. Other studies have reported similar associations between RhD negative individuals and lower HIV infection rates [17–19]. Conceivably, being RhD negative could confer some level of protection against HIV infection, or conversely the presence of RhD antigen in the host cell may act as viral receptor sites for HIV and increase the risk of infection in RhD positive individuals.

We observed no association between ABO and HIV in the overall multivariable model. A previous smaller South African study found the O RhD Negative blood group to be protective and type A RhD Positive blood group susceptible to HIV infection [20]. However, its study population was geographically limited and a lack of demographic data precluded analysis for confounding by race and other factors.

The South African population is genetically diverse and blood group distribution among different population groups can vary significantly [21]. Our study overcomes these limitations by studying a large, geographically diverse population and including demographic information to address possible confounding factors. The study by Neil *et al* [8] infers that HIV infectivity is oriented to the matching or mismatching ABO phenotype of the infecting virus and the host cell. Hypothetically, this would be best observed in populations with greater ABO blood type heterogeneity. For example, if the primary and subsequent host are both of the same blood type, the infecting virus would have the same ABO phenotype of its primary host and there would be no antibody neutralization effect. This phenomenon may also point to other unexplored immunogenetic differences related to the ABO blood group and HIV infection in populations of mixed ancestry / genetic diversity [15].

One of the limitations of our study is that it could not determine the ABO blood type of the primary host. A direct comparison of the ABO blood group antigens expressed by the infecting virus with that of the new host would yield a more accurate observation of whether neutralization by naturally occurring ABO antibodies plays a protective role in HIV infectivity in vivo. We did not include repeat blood donors in this study because our study focused on HIV prevalence. Future studies could endeavour to observe incident HIV infections in ABO-heterogeneous repeat donors and obtain biospecimens for virologic analysis. The newly produced viruses would exhibit the ABO phenotype of the primary host upon first infecting the subsequent host. Once viral replication was established in the new host, budding virions would begin to exhibit the ABO phenotype of the new host and the inhibiting effect of neutralising antibodies would be ineffective [22]. Computational models could also be used to predict the extent of ‘ABO Interference’ according to the blood group frequencies within a population, as described by Ellis [22].

In conclusion, these findings suggest that ABO blood group polymorphisms may not play a role in susceptibility to HIV infection. However, the question might benefit from further investigation using experimental models that could directly observe the ABO type of transmitter and recipient pairs. The phenomenon observed with RhD phenotype is most likely due to residual confounding.
